# Exploring the Gas-Permeation Properties of Proton-Conducting Membranes Based on Protic Imidazolium Ionic Liquids: Application in Natural Gas Processing

**DOI:** 10.3390/membranes8030075

**Published:** 2018-09-05

**Authors:** Parashuram Kallem, Christophe Charmette, Martin Drobek, Anne Julbe, Reyes Mallada, Maria Pilar Pina

**Affiliations:** 1 Department of Chemical & Environmental Engineering, Institute of Nanoscience of Aragon, University of Zaragoza, Edif. I+D+i, Campus Rio Ebro, C/Mariano Esquillor, 50018 Zaragoza, Spain; parshukallem@gmail.com (P.K.); rmallada@unizar.es (R.M.); 2 IEM (Institut Européen des Membranes), UMR 5635 (CNRS-ENSCM-UM), Université de Montpellier, CC047, Place Eugène Bataillon, 34095 Montpellier, France; Christophe.Charmette@univ-montp2.fr (C.C.); martin.drobek@univ-montp2.fr (M.D.); anne.julbe@univ-montp2.fr (A.J.); 3 School of Earth Sciences and Environmental Engineering, Gwangju Institute of Science and Technology (GIST), 261 Cheomdangwagi-ro, Buk-gu, Gwangju 61005, Korea; 4 Networking Research Center on Bioengineering, Biomaterials and Nanomedicine, CIBER-BBN, 50018 Zaragoza, Spain

**Keywords:** protic imidazolium ionic liquids, CH_4_ solubility, nanoporous polybenzimidazole membranes, supported ionic liquid membranes, photo-assisted polymerization, CH_4_ selective membranes

## Abstract

This experimental study explores the potential of supported ionic liquid membranes (SILMs) based on protic imidazolium ionic liquids (ILs) and randomly nanoporous polybenzimidazole (PBI) supports for CH_4_/N_2_ separation. In particular, three classes of SILMs have been prepared by the infiltration of porous PBI membranes with different protic moieties: 1-H-3-methylimidazolium bis (trifluoromethane sulfonyl)imide; 1-H-3-vinylimidazolium bis(trifluoromethane sulfonyl)imide followed by in situ ultraviolet (UV) polymerization to poly[1-(3H-imidazolium)ethylene] bis(trifluoromethanesulfonyl)imide. The polymerization process has been monitored by Fourier transform infrared (FTIR) spectroscopy and the concentration of the protic entities in the SILMs has been evaluated by thermogravimetric analysis (TGA). Single gas permeability values of N_2_ and CH_4_ at 313 K, 333 K and 363 K were obtained from a series of experiments conducted in a batch gas permeance system. The results obtained were assessed in terms of the preferential cavity formation and favorable solvation of methane in the apolar domains of the protic ionic network. The most attractive behavior exhibited poly[1-(3H-imidazolium)ethylene]bis(trifluoromethanesulfonyl)imide polymeric ionic liquid (PIL) cross-linked with 1% divinylbenzene supported membranes, showing stable performance when increasing the upstream pressure. The CH_4_/N_2_ permselectivity value of 2.1 with CH_4_ permeability of 156 Barrer at 363 K suggests that the transport mechanism of the as-prepared SILMs is solubility-dominated.

## 1. Introduction

The demand for natural gas (NG) is growing worldwide and there is a rising need to develop methods for upgrading sub-quality gas reserves, which exist in relatively low quantities in remote zones. The global utilization of NG is above 3.1 trillion cubic meters (110 trillion standard cubic feet) each year. NG upgrading is certainly one of the most challenging industrial applications for gas separation membranes. In fact, 14% of U.S. NG resources comprise N_2_ in significant amounts and cannot be shipped to the national pipeline without preliminary treatment. Hence, removal of this N_2_ could allow access to an estimated 10 trillion scf (standard cubic feet per day) additional NG in the USA alone [1,2,3].

So far, only a few studies on N_2_ removal from methane mixtures have been published. Membrane-based N_2_ separation has a promising market in small natural gas operations, where cryo-genic distillation is uneconomical. In general, glassy polymers are permeable to N_2_, while the rubbery ones are to CH_4_ [2]. For a gas mixture containing 10% N_2_ in CH_4_, a membrane with a N_2_/CH_4_ selectivity of at least 17 is required to achieve attractive separation in a single stage. However, the best N_2_-selective membrane currently known has a selectivity of 12.5 and permeability of 0.8 Barrer [4]; i.e., far below the attractive values. Hence, this is why the CH_4_-selective membranes are usually preferred. A process involving a CH_4_ selective membranes process remains the most feasible. Although considerable recompression of the permeate gas is required for gas delivery to the pipeline [2], its cost does not significantly impact on the process economics [1]. For a gas mixture containing 10% N_2_ in CH_4_, membrane-based separation becomes cost-effective for CH_4_/N_2_ selectivity values above 6 [3]. However, the best CH_4_-selective membrane (Polyamide-polyether copolymer-PEBAX 2533) currently known has a CH_4_/N_2_ selectivity of 4.2 and relatively low CH_4_ permeability values, i.e., 20 Barrer.

Typically, supported ionic liquid membranes (SILMs) have been extensively studied for CO_2_ separation [5,6,7,8], thanks to their good CO_2_ solubility and negligible vapor pressure; although few studies have also focused on NG upgrading [9]. In general, the possible displacement of the liquid phase in SILMs is strongly diminished and more stable membranes are obtained due to both high ionic liquids (ILs) viscosity and strong capillary forces between the IL and the supporting membrane [8,10]. The most commonly used ILs are composed of imidazolium (IM) or pyridinium (Py) cations containing one or more alkyl groups, because of their low melting points and stability under a wide range of experimental conditions. Commonly used anions include halogen atoms [11], such as tetrafluoroborate [BF_4_]^−^, hexafluorophosphate [PF_6_]^−^, and bis(trifluoromethylsulfonyl)imide [TFSI]^−^. Previous publications on SILMs confirm that the selectivity is solubility-dominated instead of diffusion-dominated [9]. The solubilities of CO_2_, CH_4_, C_2_H_6_, N_2_ and O_2_ in several aprotic ILs have been studied extensively [12,13,14,15]. On the contrary, the thermodynamic properties of protic ionic liquids, i.e., those comprising proton-donor and proton-acceptor centers in their molecules, have been investigated in lower extent [16,17,18,19].

Supported poly-ionic liquids (PILs) membranes based on protic imidazolium moieties have attracted great attention over the last decade as solid state flexible electrolytes because of their proton conductivity and superior thermal and chemical stability [20,21,22,23,24]. The main objective of this work is the exploration of the SILMs based on protic imidazolium ILs for potential CH_4_ separation applications. Among the large diversity of ILs, those based on the TFSI anion with imidazolium cation typically confer high CH_4_ permeability [25,26]. So far, all the reported SILMs for gas permeation studies have been prepared from aprotic ILs [7,27,28,29]. Unlike in the literature, our approach relies on the use of protic ILs i.e., imidazolium cation without any alkyl group at position 1 (R–N) but with acidic “H” (H–N).

Herein, we report for the first time usage of SILMs based on protic imidazolium ILs supported on/in randomly nanoporous polybenzimidazole (PBI) for gas separation of apolar compounds, i.e., CH_4_ and N_2_. The porous PBI employed for membrane fabrication as the mechanical support provides outstanding thermal and chemical stability [30]. In general, PBI exhibits very low gas permeability because of the carbon chain rigidity and strong intermolecular hydrogen bonding leading to dense packing structures [31,32]. More specifically, three classes of SILMs containing: (i) 1-H-3-methylimidazolium bis(trifluoromethane sulfonyl)imide (denoted as RPBI-IL); (ii) 1-H-3-vinylimidazolium bis(trifluoromethane sulfonyl)imide (denoted as RPBI-MIL); and (iii) poly[1-(3H-imidazolium)ethylene] bis(trifluoromethanesulfonyl)imide (denoted as RPBI-PIL) have been prepared. The RPBI-PIL family, obtained by the ultraviolet (UV) polymerization of the RPBI-MIL set, has been studied to improve the membrane’s long-term performance [33]. Indeed, IL leaching from the pores at either high temperatures or transmembrane pressures might clearly inhibit the practical use of such membranes in gas separation processes. In addition, the polymerization phase transition from liquid to solid state effectively improves the stability of the IL-based membranes [10,33,34]. Thus, a comprehensive physicochemical and single gas permeation characterization of such SILMs has been accomplished in this work. Particular emphasis is devoted to the analysis of methane solubility and to the influence of the protic cationic moieties on the gas permeability values.

## 2. Methods

### 2.1. Chemicals

All chemical reagents and solvents were used as received: Poly[2,2-(m-phenylene)-5,5bibenzimidazole] (PBI Fumion APH Ionomer, Mw = 59,000–62,000, Fumatech), LiCl (99 wt%, Sigma-Aldrich), Poly(vinylpyrrolidone) K30 (PVP K30 Mw = 40,000, Fluka), Poly(vinylpyrrolidone) K90 (PVP K90 Mw = 360,000, Fluka), 1-H-3-methylimidazolium bis(trifluoromethane sulfonyl)imide (99.5 wt%, Solvionic), 1-H-3-vinylimidazolium bis(trifluoromethane sulfonyl)imide (99.5 wt%, Solvionic), Divinylbenzene (80.0 wt%, Sigma-Aldrich), 2-hidroxy-2-methylpropiophenone (97.0 wt%, Sigma-Aldrich), *N*-methyl-2pyrrolidone (NMP anhydrous, 99.5 wt%, Sigma-Aldrich).

### 2.2. Polymer Solution Preparation

PBI was used as a polymer for the fabrication of membrane support. Polymer solutions were prepared according to a recipe previously developed in our group [21]. 11.5 g of PBI powder, 1.5 g of LiCl, 1.5 g of PVP K30, 1.5 g of PVP K90 and 84 g NMP were mixed at 448 K for 24 h to obtain 16 wt% of solids in homogeneous polymer solution. The polymer solution was then degassed under moderate vacuum for two hours to ensure that all air bubbles were removed from the solution. Addition of PVP controls macrovoids formation upon phase separation process and LiCl stabilizes the polymer solution. Before use, the PBI solution was filtered by pressurized air through metal filter (25 m in pore size) to remove insoluble solids from the starting PBI powder.

### 2.3. Preparation of the Randomly Porous Polybenzimidazole (RPBI) Supports by Phase Inversion

A schematic overview of the phase inversion process is depicted in Figure S2 (supplementary materials). The polymer solution consisting of PBI, PVP, LiCl and NMP was poured onto a clean glass plate (Figure S2A) and cast using a casting knife with a thickness of 0.25 mm. After casting, the glass plate with deposited polymer layer was immersed in a coagulation bath (Figure S2B) containing a mixture of NMP/water (50/50) for 30 min at room temperature (RT ~298 K). Then the plate was transferred into a non-solvent bath (pure water) at room temperature (RT) to wash out any NMP traces (Figure S2C), the exchange of solvent by water was effective after 30 min at RT. The polymer film was then peeled off from the plate. Subsequently solidified RPBI support was immersed in ethanol for 30 min, followed by an immersion in hexane for 30 min, to ensure complete water removal. Finally, remaining volatiles were evacuated at 423 K in an oven. For this thermal treatment, the polymer film was sandwiched between two glass plates. 

### 2.4. Fabrication of Supported Ionic Liquid Membranes (SILMs)

**Figure 1:**
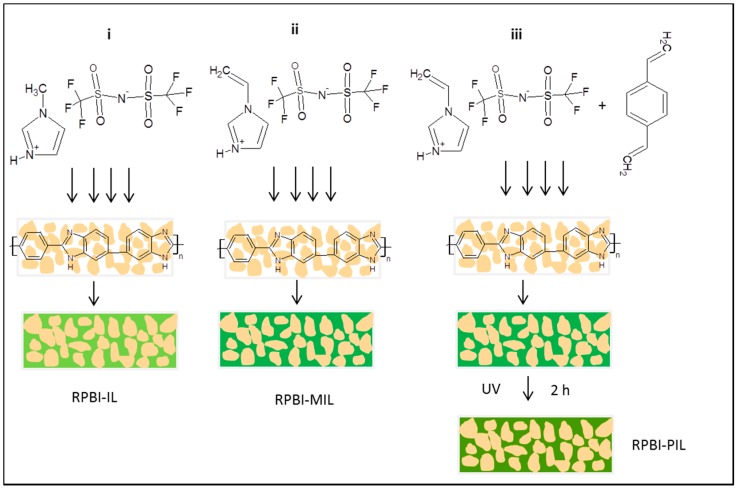
Schematic illustration of polybenzimidazole (PBI) support pore filling and chemical structures of the used ionic liquids: (i) 1-H-3-methylimidazolium bis(trifluoromethane sulfonyl)imide (H-MIM TFSI); (ii) 1-vinyl-3H-imidazolium bis(trifluoromethane sulfonyl)imide (H-VIM TFSI); (iii) H-VIM TFSI with divinylbenzene followed by polymerization with UV light.

The RPBI supports were infiltrated with ionic liquids using a protocol previously developed by the authors [21]. A schematic illustration of the infiltration protocol is shown in Figure 1. Firstly, the RPBI support was dried at 393 K under 100 mbar of vacuum to remove any water and organics. Three types of SILMs have been fabricated:

(i) RPBI-IL: the protic ionic liquid (IL) 1-H-3-methylimidazolium bis(trifluoromethane sulfonyl)imide (H-MIM TFSI) was heated up to 328 K to melt the salt. Subsequently, the RPBI support was placed under vacuum for 1h to remove air from the pores and guarantee an efficient and uniform (in)filtration of the IL through the RPBI support. The infiltration process was conducted by pouring the IL on the RPBI support surface at 443 K and applying 160 mbar vacuum. After the filtration step, the membrane was removed from the filter holder and the excess of IL on the membrane surface was wiped off with a tissue.

(ii) RPBI-MIL: the monomeric ionic liquid (MIL) 1-vinyl-3H-imidazolium bis(trifluoromethane sulfonyl)imide (H-VIM TFSI) was heated up to 323 K to melt the salt, and the above (in)filtration protocol was applied.

(iii) RPBI-PIL: the monomeric ionic liquid (MIL) 1-vinyl-3H-limidazolium bis(trifluoromethane sulfonyl)imide was heated up to 323 K to melt the salt; afterwards 1 mol% (referred to the MIL) of divinylbenzene (crosslinker-CL) was added and the mixture was thoroughly mixed. Subsequently, the above (in)filtration protocol was applied. Finally, a photo initiator (2-hydroxy-2-methylpropiophenone) was added on the membrane top-surface to initiate photo-polymerization. In order to obtain the crosslinked RPBI/PIL composite membranes, each side of the membrane surface was exposed for 2 h under a 365 nm UV lamp (Vilber Lourmat, Collégien, France) with intensity of 2.4 mW cm^−2^). After IL polymerization, the membrane surface was gently wiped from any residuals with a lab paper.

### 2.5. Characterization Methods

**Porosity:** The porosity of the as prepared RPBI support was determined by using a helium displacement pycnometer (Micromeritics AccuPyc 1330, Micromeritics Instrument Corp., Norcross, GA, USA) equipped with 1 cm^3^ sample module. The reported porosity values were obtained for RPBI supports with more than 50 cm^2^ surface area. Porosity was calculated using following equation:

(1) $$\mathrm{Porosity}\;\left(\emptyset \right)=\frac{\mathrm{Vbulk}-\mathrm{Vskeleton}}{\mathrm{Vbulk}}\;\times \;100\%\;$$

where Vbulk is directly estimated from surface area and thickness of the RPBI sample and Vskeleton is obtained from the instrument. 

For all samples, measurement reproducibility was typically within ±0.01% of the nominal porosity value.

**Scanning electron microscopy (SEM) characterization:** the morphology, thickness, porous structure and pore size of the as-prepared RPBI supports were observed by scanning electron microscopy (SEM) (FEI INSPECT 50), acceleration voltage 15 keV. Prior to observation, the samples were coated with a Pd layer of ca. 2 nm by sputtering (LEICA EM ACE200).

**Transmission electron microscopy (TEM):** membranes were embedded in epoxy resin, and ultrathin slices (about 50 nm thick) were cut with an ultramicrotome (Leica EM UC7) at room temperature. These slices were placed on TEM copper grids with carbon film, and analyzed by TEM in a Tecnai T20 (FEI Company), at a working voltage of 200 KV. TEM bright field images were acquired with a side-mounted Veleta CCD Camera.

**Atomic force microscopy (AFM):** AFM measurements have been carried out by tapping mode using NSG30 ND-MDT tip (Multimode 8 system, Veeco/Bruker) with force constant around 22–100 N/m. Roughness average (Ra) and root mean square (RMS) values are both representations of surface roughness, although calculated differently from microscopic peaks and valleys on the surface using the following equations:

(2) $$\;Ra=\frac{1}{N}\overset{n}{\underset{i=1}{\sum }}\;\left[yi\right]\;$$

(3) $$\;RMS=\sqrt{\frac{1}{N}\overset{n}{\underset{i=1}{\sum }}yi²}\;$$

The roughness profile contains *N* ordered, equally spaced points along the trace, and *yi* is the vertical distance, expressed in nm, from the mean line to the *i*th data point.

**Infrared spectra measurements:** attenuated total reflection–Fourier transform infrared (ATR-FTIR) analyses (Bruker VERTEX 70 equipped with Golden Gate ATR from 4000 to 600 cm^−1^, 256 scans and resolution of 4 cm^−1^) were performed at room temperature to assess about the photo-polymerization evolution in RPBI/PIL SILMs, and to investigate any possible interactions between the benzimidazole from the RPBI support and the poly[1-(3H-imidazolium)ethylene] bis(trifluoromethane sulfonyl)imide.

**Thermogravimetric studies:** thermogravimetric analyses (TGA) were carried out using a Q500 IR TA instrument to evaluate the composition and thermal behavior of the as-prepared SILMs. Studies were conducted using 4–5 mg samples, in the temperature range from room temperature up to 1173 K at a controlled heating rate of 2 K/min under an inert atmosphere (N_2_). 

**Methane solubility in the protic ionic liquid:** the CH_4_ gas solubility in the H-VIM TFSI was calculated by using the experimental vapour pressure equilibrium. The vapor pressure of the protic ionic liquid mixture with methane was measured at 333 K at five compositions (from 0.0056 to 0.0165 methane molar fraction) in the experimental set-up described by Coronas et al. [35] using a static isochoric method. 

**Single gas permeation experiments:** single gas permeation measurements through the membranes were carried out by using the constant-volume and variable-pressure technique at controlled temperature, as described in the standard ASTM D 1434-82 protocol (procedure V). A schematic of the experimental set-up (home-made) is shown in Figure S3. The two compartments of the permeation cell are separated by the tested membrane. The permeability was obtained by measuring the pressure increase in the downstream compartment (with a constant volume of 5.25 10^−5^ m^3^) and using different MKS Baratron pressure transducers (range from 0.0 to 1 × 10^5^ Pa). The membrane and downstream cell walls were initially outgassed in situ during 15 h at high vacuum using a turbomolecular pump (Leybold, Turbovac 50). Permeability values were measured in the temperature range from 313 K to 363 K, using classically up to 1.5 × 10^5^ Pa of upstream pressure gauge (unless otherwise indicated). The pressure increase in the downstream compartment was continuously measured during 4 h. For each temperature change, the whole set-up was stabilized during at least 12 h. For a given temperature, the order of gas permeance measurements was as follows: N_2_, CH_4_. Between each measurement, both the membrane and the cell were outgassed in situ during 12 h under high vacuum.

Both N_2_ and CH_4_ were provided by Linde Gas as single gases with 99.95% purity, and were used without any further purification. A complete description of the experimental system and measurement protocol was reported elsewhere [36]. For permeability calculations, a mathematical treatment relevant for thin films and based on the second Fick’s law was used:

(4) $$\;P=\frac{V\;L}{A\;R\;T\;P_{1}\;}\left(\frac{{\mathrm{dP}}_{2}}{\mathrm{dt}}\right)\;$$

where P (mol m^−1^ s^−1^ Pa^−1^) is the gas permeability; V (m^3^) is the volume of the downstream compartment; L (m) is the membrane thickness; A (m^2^) is the membrane surface area; R is the universal gas constant (Pa m^3^ mol^−1^ K^−1^); T is the permeation temperature (K); P_1_ (Pa) is the applied feed side pressure; and P_2_ (Pa) is the recorded pressure at the permeate side.

## 3. Results and Discussion

### 3.1. Fabrication of the Randomly Porous Polybenzimidazole (RPBI) Supports: Morphological Characterization

**Figure 2:**
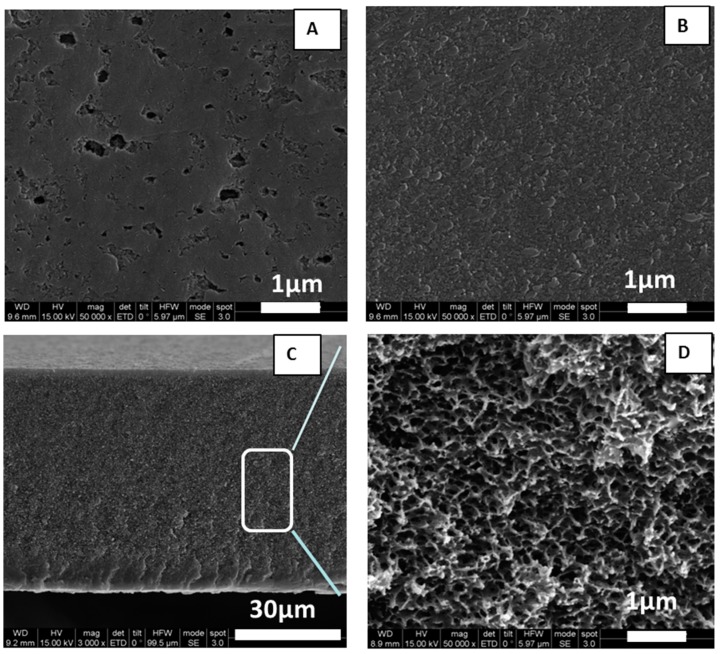
Scanning electron microscope (SEM) observation of a RPBI support prepared by phase separation method from 16 wt% of solid in the polymer solution: (**A**) air (top) side; (**B**) glass (bottom) side; (**C**) cross-section; (**D**) detail of cross-section area.

The RPBI supports, 120 to 175 μm thick, were prepared successfully by a phase separation method already reported in our previous work [21]. The porosity measured by pycnometry was 63.7 ± 2.7%. SEM pictures of the prepared RPBI supports are shown in Figure 2, where the analysis of airside, glass side and cross-section are displayed. Pore sizes in the range 50–250 nm were measured on the air side and 30–50 nm on the glass side. The cross-section view reveals a sponge-like structure.

**Figure 3:**
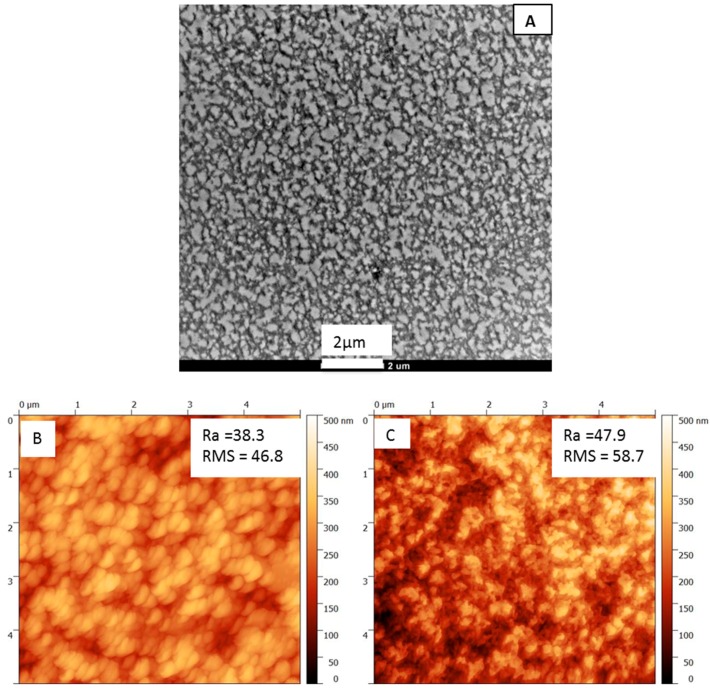
(**A**) Transmission electron microscope (TEM) observation of randomly porous PBI (RPBI) support and AFM surface images of (**B**) RPBI-glass (bottom) side and RPBI-air (top) side (**C**). The values of roughness average (Ra) and root mean squared (RMS) roughness, expressed in nm, are reported on the atomic force microscope (AFM) images.

To better understand the pore connectivity, the microstructure of RPBI support was observed by TEM. A typical image of the cross-section is shown in Figure 3A. The clear regions correspond to the pores and interconnections between random pores can be observed over the whole membrane thickness. In order to examine the surface roughness of the RPBI support, AFM surface images of both glass and air side (bottom and top side, respectively) were analyzed (Figure 3B,C). The minor changes in roughness parameters (roughness average and root mean squared roughness values reported on the AFM images) from top to bottom are attributed to the change in the size of interconnected open pores.

### 3.2. Fabrication of SILMs Based on Protic Imidazolium Ionic Liquids: Physico-Chemical Characterization

SILMs were prepared by infiltration of RPBI support with the protic ionic liquids, 1-H-3-methylimidazolium bis(trifluoromethane sulfonyl)imide monomeric ionic liquid (H-MIM TFSI) and 1-H-3-vinylimidazolium bis(trifluoromethane sulfonyl)imide (H-VIM TFSI), respectively, as described in the experimental section. Due to the viscosity of H-MIM TFSI (i.e., 80 cP at 298 K) [22] and H-VIM TFSI (14.3 cP at 323 K), the use of both vacuum and high temperature was required to ensure efficient IL embedding within the pores of the RPBI support. A schematic illustration of the infiltration protocol is shown in Figure 1A.

**Figure 4:**
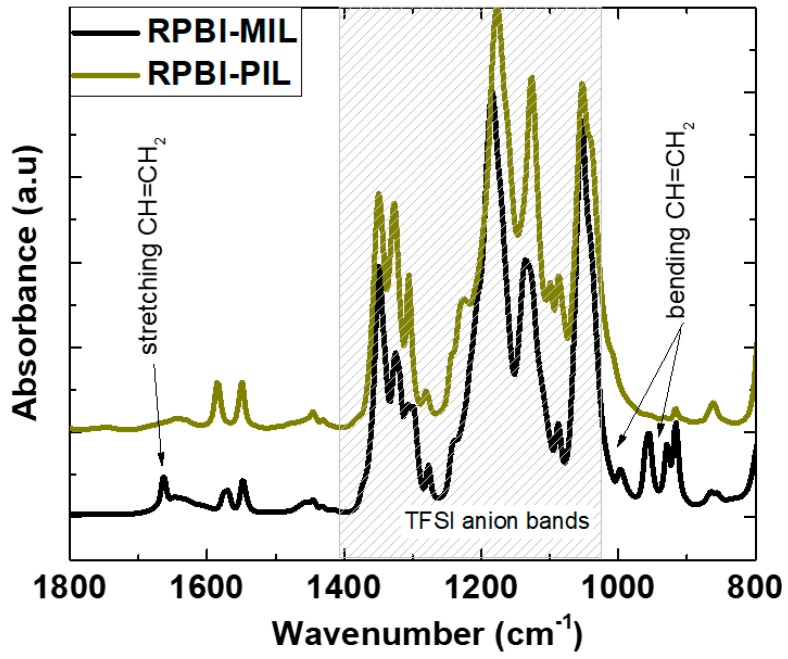
Attenuated total reflection–Fourier transform infrared (ATR-FTIR) spectra of the resulting SILM membrane before and after UV irradiation.

FTIR analyses were used to evidence the successful polymerization of vinyl-polymerizable groups. Accordingly, ATR-FTIR spectra of the composite membranes before (RPBI-MIL), and after 2 h UV irradiation (RPBI-PIL) are compared in Figure 4. The intense absorption bands in the range 1400–1000 cm^−1^, observed for both RPBI-MIL and RPBI-PIL membranes, are characteristic of the –SO_2_– and –SNS– vibrational modes of the bis(trifluoromethanesulfonyl)imide [TFSI] anion [37]. Two characteristic infrared absorbance bands in RPBI-MIL were selected to examine the disappearance of the vinyl-monomer: 1665–1630 cm^−1^ (stretching vibration in –CH=CH_2_) and 995–920 cm^−1^ (out of plane bending of –CH=CH_2_ groups). The disappearance of these characteristic peaks in RPBI-PIL upon 2 h UV light exposure confirmed a successful polymerization, above 97%, as already demonstrated in our previous studies [20,21,23,24]. Figure S1 (supplementary material) shows photos of the prepared SILMs, as free-standing films. The SILMs based on IL (i.e., RPBI-IL) were extremely brittle and hard to handle, due to the IL crystallinity at room temperature (melting point ~328 K) whereas the RPBI-MIL was only slightly brittle when handling (melting point ~313–318 K). Contrary, the SILMs based on polymeric IL (i.e., RPBI-PIL) were very easy to manipulate.

Table 1 summarizes the characteristics of the as prepared SILMs. The experimental IL/MIL/PIL loadings calculated from simple weight increase measurements reasonably match those evaluated from TGA (accounting from the registered weight loss within the 473–778 K temperature range), but overpass theoretical values due to the difficulty in wiping the excess of IL/MIL/PIL from the membrane surface. Therefore, the infiltration process herein performed ensures complete pore filling of the PBI supports. 

**Figure 5:**
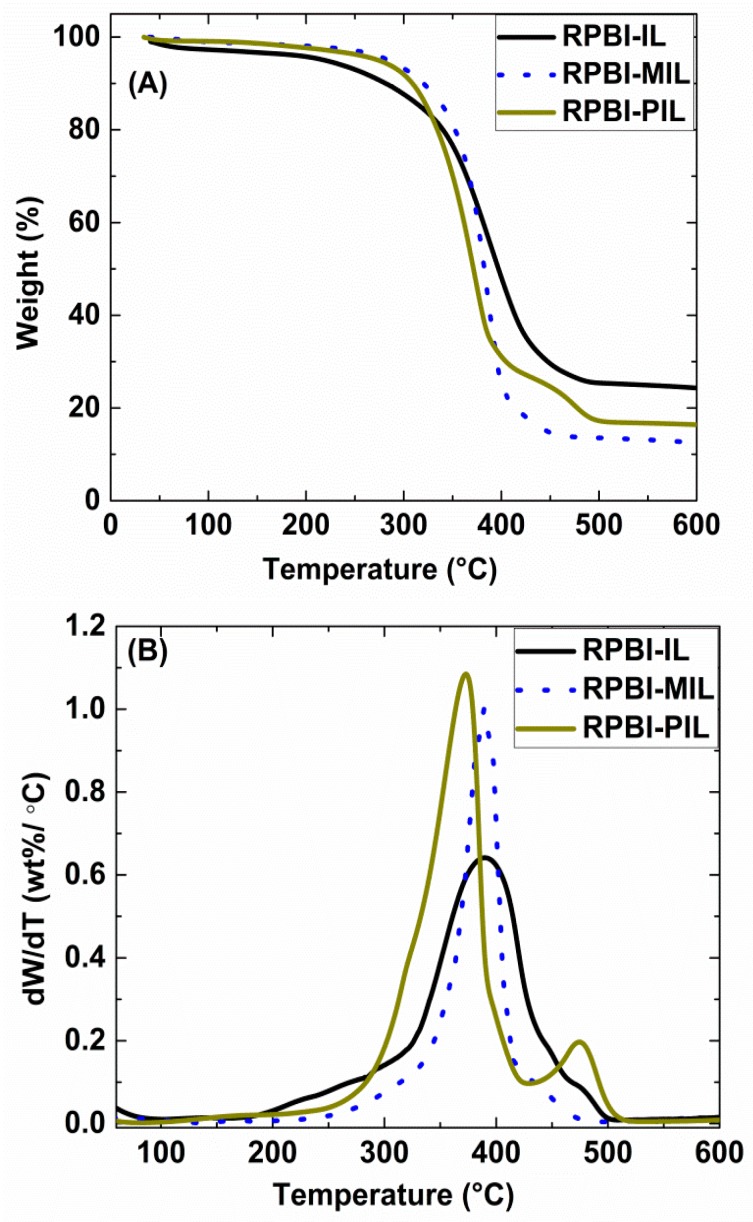
(**A**) Thermogravimetric analysis (TGA) curves and (**B**) derived differential (DTG) curves of the prepared supported ionic liquid membranes.

Figure 5 shows the TGA and derived DTG curves of all the prepared SILMs. A one stage thermal decomposition process (663 K) was observed for both RPBI-IL and RPBI-MIL samples due to the decomposition of H-MIM TFSI and H-VIM TFSI, respectively. Whereas in the case of RPBI-PIL membrane a two-stage decomposition was observed: the first weight loss corresponds to PIL decomposition and the shoulder at ~753 K is attributed to interactions between PIL and RPBI support [21]. Very low weight loss (0.7–1.5%) were measured for all SILMs within the 423–473 K temperature range, suggesting a good thermal stability of SILMs up to 583 K under N_2_ atmosphere. The measured Young’s modulus and tensile strength values of RPBI-PIL were 0.2 GPa and 1.3 MPa, respectively (refer to our previous work [23] for more details). The RPBI-IL and RPBI-MIL samples were not considered for mechanical tests due to handling difficulties at room temperature.

### 3.3. Permeation Properties of the SILMs Based on Protic Imidazolium Ionic Liquids

To the best of our knowledge, the permeation properties of SILMs based on protic imidazolium ionic liquids have been scarcely investigated in the literature. In this work, the single gas permeances of N_2_, CH_4_ were measured in order to evaluate the membrane permselectivity (PermSel CH_4_/N_2_) for CH_4_/N_2_ separation which is an important parameter for possible upgrading of natural gas.

In parallel, solubility data of gases in ionic liquids are required for designing the separation processes and provide the basis for tuning the ionic liquids properties. The potential of ionic liquids for the separation of CH_4_/N_2_ gas mixture can be evaluated by the ideal selectivity (Ideal Sel CH_4_/N_2_) which is defined by the ratio of Henry’s constant values (H N_2_/H CH_4_).

Table 2 compares the Henry’s constant values for CH_4_ and N_2_ at different temperatures in the range 298–343 K, in common aprotic ionic liquids based on methylimidazolium cations and [TFSI] anion. CH_4_ is the most soluble (lowest Henry’s constant), while the solubility of N_2_ is lower (higher Henry’s constants) for all the tested conditions. Regular solution theory has been extensively used as a method to model the behavior of gases in aprotic ILs. The widely investigated CO_2_ + IL system could be accurately modeled as a function of the sorbent molar volume, with smaller molar volumes and lower temperatures yielding both increasingly higher CO_2_ solubilities and ideal CO_2_/gas selectivities.

Table 2 displays the solubility selectivity trend for CH_4_ and N_2_ pairs as a function of temperature. The same solubility selectivity trends exist for all the aprotic ionic liquids tested. With increasing temperature, the solubility selectivity slightly decreases, i.e. from 2.9 at 298 K to 1.7 at 343 K. This behavior was expected when considering the observed evolution trend of solubility vs. temperature. Unlike the CO_2_ + ILs equilibria behaviors, the solubility of N_2_ increases (decreasing Henry’s constant) when temperature increases for all the aprotic ionic liquids. On the other hand, the CH_4_ solubility remains almost constant, indicating that the change in partial molar enthalpy and entropy of the system must be zero (based on thermodynamic equations). Additionally, the Henry´s constant value for CH_4_ decreases when the molar volume of the aprotic IL increases, i.e., from 580 atm to 350 atm at 298 K for 1-ethyl-3-methylimidazolium bis(trifluoromethanesulfonyl)imide and -hexyl-3-methylimidazolium bis(trifluoromethanesulfonyl)imide, respectively. When comparing the ideal selectivity values obtained from solubility measurements (Sel CH_4_/N_2_) with those corresponding to membrane permselectivity (Perm Sel CH_4_/N_2_), a solubility dominated transport was confirmed for aprotic ionic liquids [9].

Table 3 compares the Henry´s constant values for CH_4_ and N_2_ in the temperature range 303–333 K, in protic ionic liquids, i.e., those containing proton-donor and proton-acceptor centers in their molecules. Protic ionic liquids are described as structurally heterogeneous compounds consisting of both polar and apolar domains. Charged and uncharged groups tend to segregate resulting in sponge-like nanostructures. In general, the thermodynamic properties of ionic liquids with dissociable protons are significantly less investigated than those of aprotic analogues. An inert-gas stripping method has been described in the literature [16] for measuring solubilities of moderately and sparingly soluble gases, i.e., N_2_, O_2_, air, C_2_H_4_, C_2_H_6_ in low viscosity protic ionic liquids such as 1-butyl, 3-H-imidazolium acetate. In protic ionic liquids, CH_4_ is the most soluble (lowest Henry’s constant) while the solubility of N_2_ lower (higher Henry’s constant) than in their aprotic imidazolium counterparts. The CH_4_ Henry’s constant for the 1-H-3-vinylimidazolium bis(trifluoromethanesulfonyl)imide ionic liquid used in this work is 172 ± 16 atm at 333 K, i.e., three fold lower than the values measured for the aprotic 1-ethyl-3-methylimidazolium bis(trifluoromethanesulfonyl)imide. At a first glance, the intermolecular hydrogen bonds in protic molecular solvents seem to yield a significant drop of solubility for apolar species. However, here we do observe a tendency of higher CH_4_ solubility in comparison with similar aprotic ionic liquids. Sedov et al. [17] explain this behaviour by a preferential cavity formation and favorable solvation of hydrocarbons in the apolar domain of nanostructured protic ionic liquids. Consequently, the higher CH_4_ permeability values measured for SILM_S_ prepared from protic ionic liquids would be expected.

**Figure 6:**
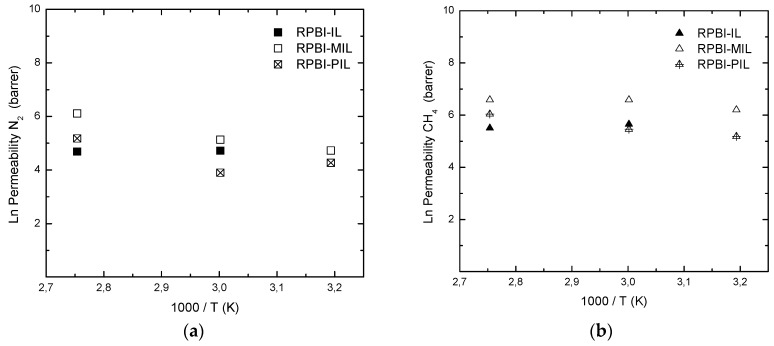
Influence of temperature on single gas permeability values for: N_2_ (**a**), CH_4_ (**b**).

**Figure 7:**
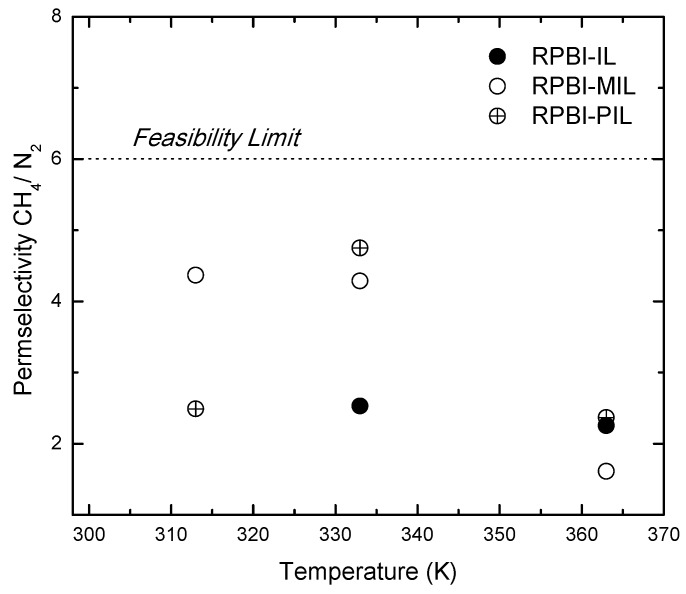
Influence of temperature on CH_4_/N_2_ permselectivity values.

In this work, the single gas permeability results were assumed to reflect gas transport through protic ionic liquid moieties while the contribution of the parallel RPBI transport pathway was considered as negligible due to the extremely low permeabilities of gases in dense PBI [39], i.e., 0.009 Barrer for CH_4_. The influence of temperature on both gas permeability (Figure 6) and CH_4_/N_2_ permselectivity (Figure 7) was studied for the three different SILMs in the temperatures range 313–363 K and measured in the initial pressure range of 1.5 barg. Since H-MIM TFSI is a crystalline solid at room temperature and its melting point is ~328 K [40], the experiments with RPBI-IL were carried out at 333 K and above.

The measured permeability values of the prepared SILMs are in the range 49–178 Barrer for N_2_ and 178–725 Barrer for CH_4_. In the tested temperature range, i.e., 333–363 K, the CH_4_ permeability of RPBI-MIL membranes is always higher than for RPBI-IL membranes. Hence, the effect of vinyl substitution on imidazolium group seems to increase the CH_4_ solubility. According to Scovazzo et al. [9], in addition to the consideration of IL viscosity and molar volume, the IL ability to accept hydrogen to form a hydrogen bond contributes to a better correlation of the permeance trends for N_2_, CH_4_ and C_x_H_y_ through SILMs. In our previous work [20] on the use of protic ionic liquids for the preparation of all solid state ion conductive films, the proton transport properties of HVIM TFSI were found to be superior to those of the HMIM TFSI counterpart. Hence, proton conduction properties seem to be in line with observed CH_4_ permeation values.

All solid-state gas permeable membranes, denoted as RPBI-PIL, were also prepared by UV photo-assisted polymerization of supported HVIM TFSI membrane to provide SILMs with adequate physical stability for gas separation applications involving moderate to high trans-membrane pressures. As expected, the cationic moieties polymerization strongly impacts the membrane permeation properties. In fact, gas permeability values of RPBI-PIL membranes were three times lower than those measured for RPBI-MIL membranes (Table 4). Above all, when compared with the RPBI-IL counterparts, the CH_4_ permeance through RPBI-PIL resembles the same at the expense of a remarkable improvement of endurance properties. 

In this study, the N_2_ permeability values tend to increase moderately within the tested temperature window; whereas CH_4_ is less temperature dependent (Figure 6), which is in a good agreement with the gas solubility data reported in Table 2. 

The calculated CH_4_/N_2_ PermSel values, corresponding to the ratio of single gas permeabilities, are reported in Table 4. As observed in Figure 7, the SILMs developed in this work exhibit relatively high PermSel values, up to 4.7 for RPBI-PIL at 333 K. Although this value is below 6, i.e., the target estimated by Baker [3] for cost-effective NG processing with membranes, the methane permeability through RPBI-PIL remains always above 60 Barrer for the tested temperature window.

**Figure 8:**
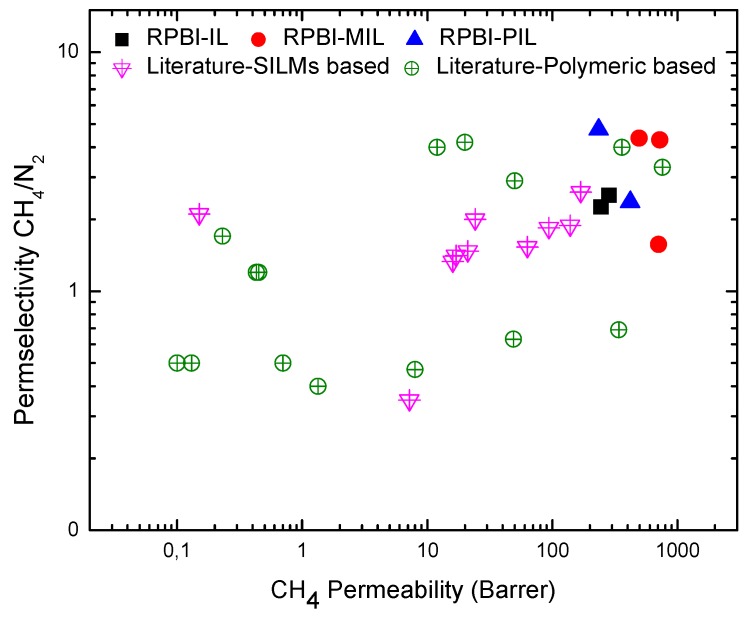
Comparison of permselectivity vs. permeability values for the membranes prepared in this work and for series of SILMs and polymer membranes reported in the literature.

The key difference between IL-based and polymer-based membranes is the impact of gas diffusivity on membrane selectivity. In IL-based membranes, the gas diffusivity selectivity is constant for a given gas pair, whereas the solubility selectivity controls membrane selectivity [21]. In most polymeric membranes, the opposite behavior is observed: solubility selectivity is usually constant for gas pairs and it is the diffusivity selectivity which determines the membrane selectivity [26,41]. The CH_4_/N_2_ permselectivity values for all the herein studied SILMs are plotted in Figure 8 as a function of CH_4_ permeability.

It is evident that the performance of the SILMs developed in this work are highly promising in comparison with literature data for either polymeric membranes (adapted from both Lokhandwala et al. [1] and Scholes et al. [2]) or other SILMs (based on aprotic imidazolium cation and TFSI anion on/in different supports) [7,25,26,27,28,29,42,43].

**Figure 9:**
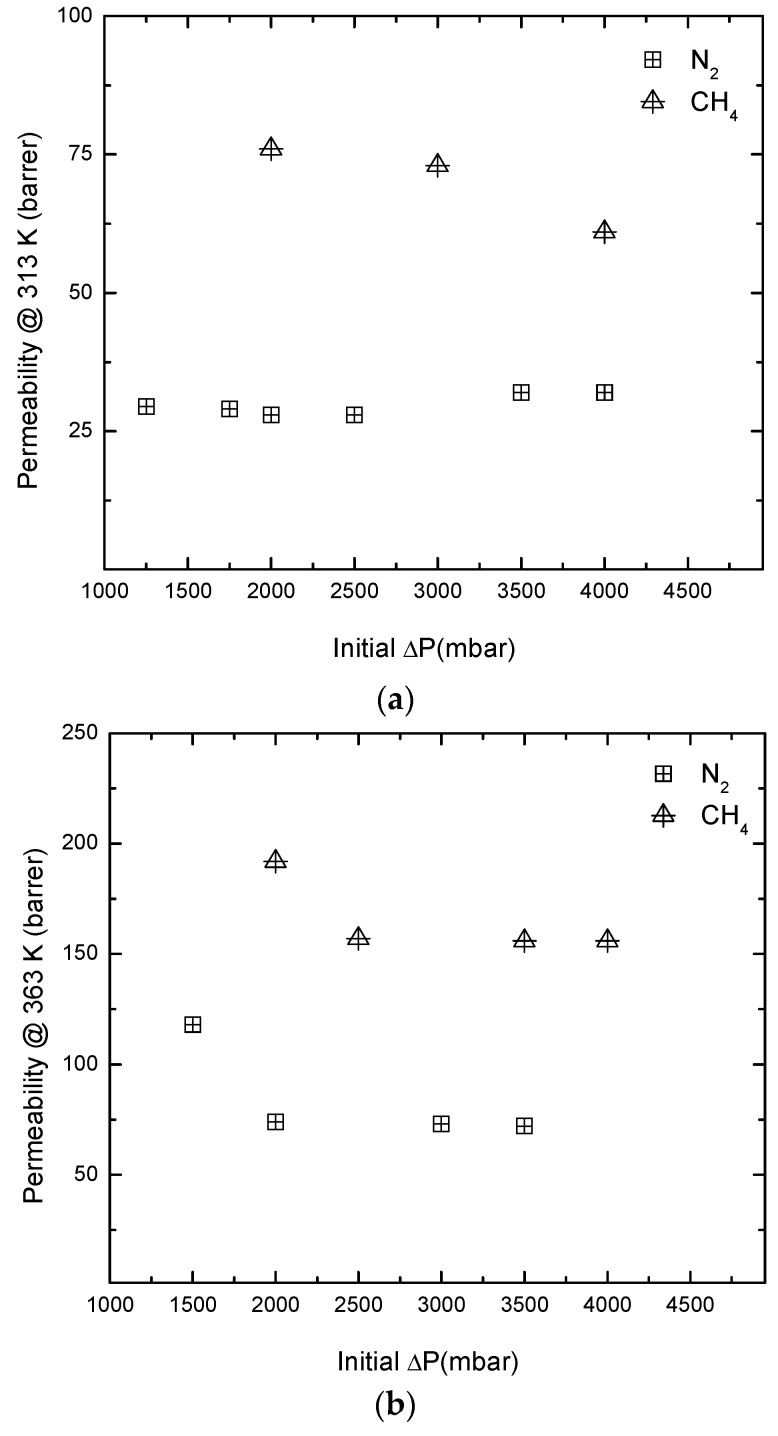
Influence of initial feed side pressure on single gas permeability values as a function of temperature: 313 K (**a**) and 363 K (**b**) for RPBI-PIL membranes.

Among all the prepared SILMs, the RPBI-PIL family stands for the most adequate in terms of CH_4_/N_2_ transport properties. In a step further, these membranes were subjected to gas permeance experiments using an initial up-stream pressure up to 4 × 10^5^ Pa for durability evaluation. Figure 9 summarizes the results obtained for both single gases at 313 K and 363 K, respectively. A slight decline in the measured CH_4_ and N_2_ permeability values was observed when the initial upstream pressure increased from 1.5 barg to 2.5 barg at 363 K. Apart from this observation, the permeation properties remain constant whatever the pressure applied in the feed side: 32 to 72 Barrer for N_2_ and 61 to 156 Barrer for CH_4_ at 313 K and 363 K, respectively. These results confirm the expected endurance provided by the polymerization of the cationic moieties in the RPBI support.

The polymerized IL, nanoconfined in the RPBI support, have been shown to provide stable performance with both relatively high CH_4_ permeability (>60 Barrer) and stable CH_4_/N_2_ permselectivity (in the range 2.0–4.7) up to 363 K and 4.0 barg, and attractive performance is also expected for the separation of gas mixtures with the RPBI-PIL membrane family upon long-term operation. This will be the subject of our future investigations, focusing in more details on the gas permeation and separation measurements for a long period of time.

## 4. Conclusions

In this work we presented for the first time an experimental study of N_2_ and CH_4_ permeation properties of supported ionic liquid membranes (SILMs) based on protic imidazolium [TFSI] ionic liquids supported in randomly nanoporous PBI (RPBI). So far, only limited studies can be found in the literature on the separation performance of bulky protic ionic liquids focusing essentially on the evaluation of the ideal CH_4_/N_2_ selectivity calculated from Henry’s constant values. Unexpectedly, the CH_4_ solubility in the 1-H-3-vinylimidazolium bis(trifluoromethanesulfonyl)imide ionic liquid used in this work is three times higher than the values measured for its similar aprotic counterparts. This observation is attributed to the favorable solvation of hydrocarbons in the apolar domains of nanostructured protic ionic liquids. 

The measured permeability values of the prepared SILMs based on 1-H-3-methylimidazolium [TFSI], 1-H-3-vinylimidazolium [TFSI] and poly[1-(3H-imidazolium)ethylene] [TFSI] were found to be in the range 49–178 Barrer for N_2_ and 178–725 Barrer for CH_4_ at temperatures varying from 313 to 363 K. Among the studied SILMs, those based on poly[1-(3H-imidazolium)ethylene] [TFSI] are clearly superior with CH_4_/N_2_ permeation properties comparable or higher than the state of the art membranes, i.e., a CH_4_/N_2_ permselectivity of 4.7 with a CH_4_ permeability reaching 235 Barrer at 333 K. The membrane permeability is above the target and particularly attractive for industrial applications.

In addition, such solid-state gas selective poly-ionic liquid-based membranes exhibit stable performance at moderate trans-membrane pressures, i.e., 4 barg, thanks to the in situ polymerization and confinement of the cationic moieties within the pores of the RPBI support. This work is a strong motivation for future investigations of poly[1-(3H-imidazolium)ethylene] [TFSI] supported membranes in a long-term performance operation with gas mixtures relevant for natural gas upgrading.

## Figures and Tables

**Table 1:** Main characteristics of SILMs based on protic imidazolium moieties specifically prepared for this work.

SILM	Ionic liquid (IL) Loading (wt%)		
	Theoretical ^1^	Experimental ^2^	TGA
RPBI-IL	73.5	82.4	70.4
RPBI-MIL	77.0	86.0	83.1
RPBI-PIL	82.5	86.5	78.6

**^1^** Theoretical calculations based on both IL/MIL/PIL density and membrane porosity; ² Experimental estimation from weight measurements.

**Table 2:** Values of Henry’s law constant for N_2_ and CH_4_ in different aprotic ILs and derived calculated ideal selectivities.

Aprotic Ionic Liquids	T (K)	H N_2_ (atm)	H CH_4_ (atm)	Ideal Sel CH_4_/N_2_	Ref.
1-hexyl-3-methylimidazolium bis(trifluoromethanesulfonyl)imide	298	1000 ± 8	350 ± 1	2.8	[14]
	313	830 ± 6	350 ± 2	2.4	
	328	720 ± 11	340 ± 4	2.1	
	343	660 ± 12	340 ± 0.4	1.9	
1-butyl-3-methylimidazolium bis(trifluoromethanesulfonyl)imide	333	970 ± 30	420 ± 10	2.3	[38]
1-ethyl-3-methylimidazolium bis(trifluoromethanesulfonyl)imide	298	1400 ± 17	580 ± 4	2.9	[14]
	313	1200 ± 27	560 ± 3	2.1	
	328	1000 ± 19	540 ± 1	1.85	
	343	910 ± 0.3	530 ± 0.4	1.7	

**Table 3:** Values of Henry’s law constant for N_2_ and CH_4_ in different imidazolium based protic ILs and derived ideal selectivity values.

Protic Ionic Liquids	T (K)	H N_2_ (atm)	H CH_4_ (atm)	Ideal Sel CH_4_/N_2_	Ref.
1-butyl-3-H-imidazolium acetate	308	1840 ± 147	90 ± 4.5 * 85 ± 3.4 **	20.4 * 21.6 **	[16]
1-H-3-vinylimidazolium bis(trifluoromethanesulfonyl)imide	333	n.a.	172 ± 16	n.a.	This work

* evaluated for C_2_H_6_; ** evaluated for C_2_H_4_.

**Table 4:** Single gas permeability values measured for the SILMs prepared in this work and derived permselectivity values.

Ionic Liquid	Support	Temperature (K)	N_2_ (Barrer)	CH_4_ (Barrer)	PermSel CH_4_/N_2_
1-H-3-methylimidazolium bis(trifluoromethane sulfonyl)imide	RPBI	333	112	285	2.5
1-H-3-vinylimidazolium bis(trifluoromethanesulfonyl)imide	RPBI	333	169	725	4.3
poly [1-(3H-imidazolium) ethylene] bis (trifluoromethanesulfonyl)imide	RPBI	333	50	235	4.7
1-ethyl-3-methylimidazolium bis(trifluoromethanesulfonyl)imide	PVDF *	303	17	32	1.9

* Data from Reference [9]: commercial PVDF 125 μm thick, 70% porosity, 0.1 m pore diameter.

## Supplementary Materials

The following are available online at http://www.mdpi.com/2077-0375/8/3/75/s1, Figure S1: Photos of the prepared SILMs: A) IL-based SILM (RPBI-IL); B) MIL-based SILM (RPBI-MIL); C) PIL-based SILM (RPBI-PIL), Figure S2: Schematic of the phase inversion steps: (A) polymer solution casting on clean glass plate; (B) System immersed in a coagulation bath with solvent mixture 50:50% of NMP: water; (C) Glass plate with the formed PBI support immersed in pure water, Figure S3: Schematic of the lab-scale experimental set-up used for single gas permeation measurements.
