# Supplementary material for: Exploring the Gas-Permeation Properties of Proton-Conducting Membranes Based on Protic Imidazolium Ionic Liquids: Application in Natural Gas Processing
Source: Membranes (Basel). 2018 Sep 5;8(3):75. doi: 10.3390/membranes8030075 (PMC6161093; doi:10.3390/membranes8030075)
Supplement: Supplementary file 1 [file membranes-08-00075-s001.pdf]

membranes

Supplementary Materials

Exploring the Gas-Permeation Properties of Proton-Conducting Membranes Based on Protic Imidazolium Ionic Liquids: Application in Natural Gas Processing

Parashuram Kallem [1,2,3], Christophe Charmette [2], Martin Drobek [2], Anne Julbe [2], Reyes Mallada [1,4] and Maria Pilar Pina [1,4,*]

1 Department of Chemical & Environmental Engineering, Institute of Nanoscience of Aragon, University of Zaragoza, Edif. I+D+i, Campus Rio Ebro, C/Mariano Esquillor, 50018 Zaragoza, Spain; parshukallem@gmail.com (P.K.); rmallada@unizar.es (R.M.)

2 IEM (Institut Européen des Membranes), UMR 5635 (CNRS-ENSCM-UM), Université de Montpellier, CC047, Place Eugène Bataillon, 34095 Montpellier, France; Christophe.Charmette@univ-montp2.fr (C.C.); martin.drobek@univ-montp2.fr (M.D.); anne.julbe@univ-montp2.fr (A.J.)

3 School of Earth Sciences and Environmental Engineering, Gwangju Institute of Science and Technology (GIST), 261 Cheomdangwagi-ro, Buk-gu, Gwangju 61005, Korea.

4 Networking Research Center on Bioengineering, Biomaterials and Nanomedicine, CIBER-BBN, 50018 Zaragoza, Spain.

* Correspondence: mapina@unizar.es; Tel. +34-976-761155

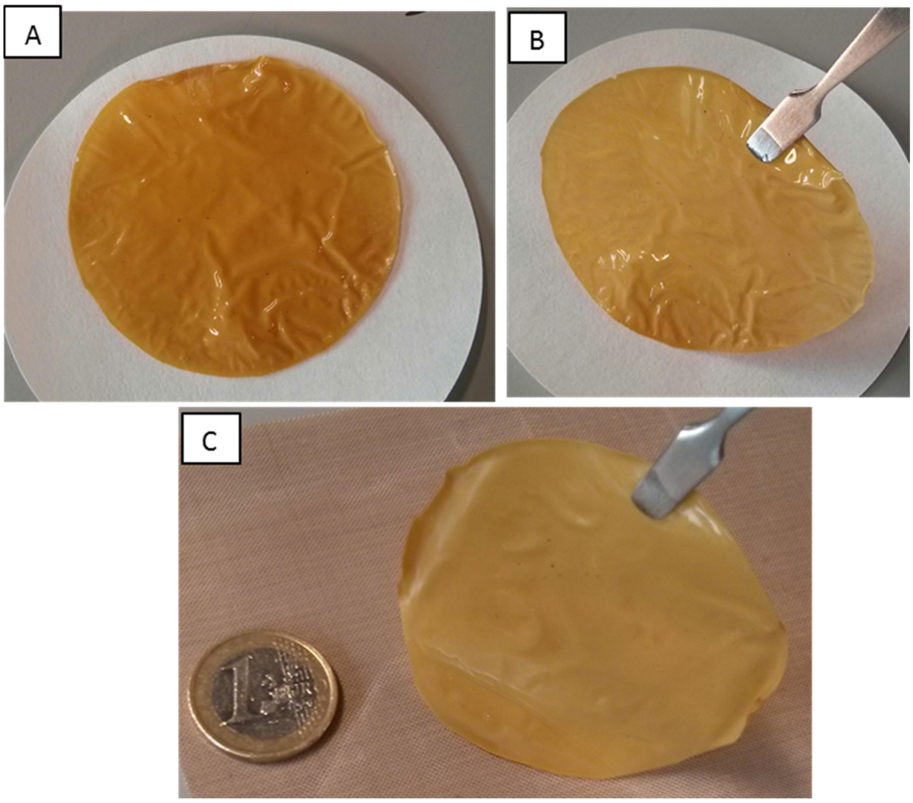

Figure S1. Photos of the prepared SILMs: A) IL based SILM (RPBI-IL); B) MIL based SILM (RPBI-MIL); C) PIL based SILM (RPBI-PIL).

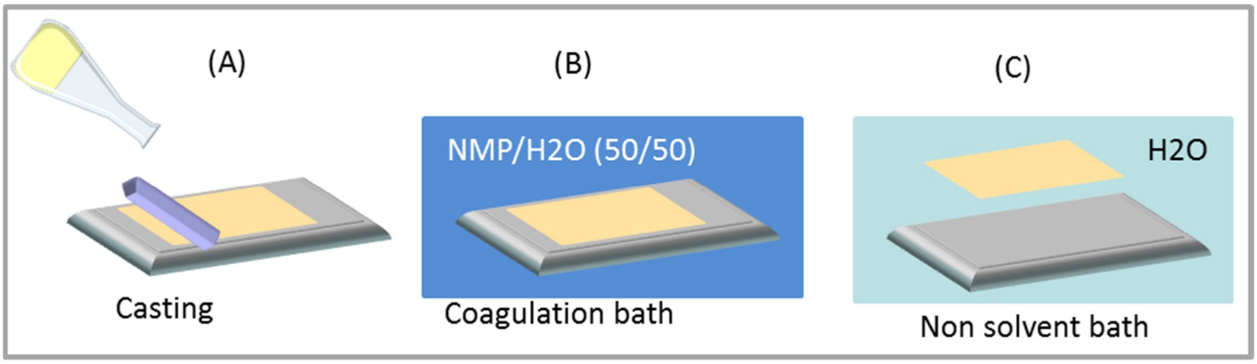

Figure S2. Schematic of the phase inversion steps: (A) polymer solution casting on clean glass plate; (B) System immersed in a coagulation bath with solvent mixture 50:50% of NMP: water; (C) Glass plate with the formed PBI support immersed into pure water.

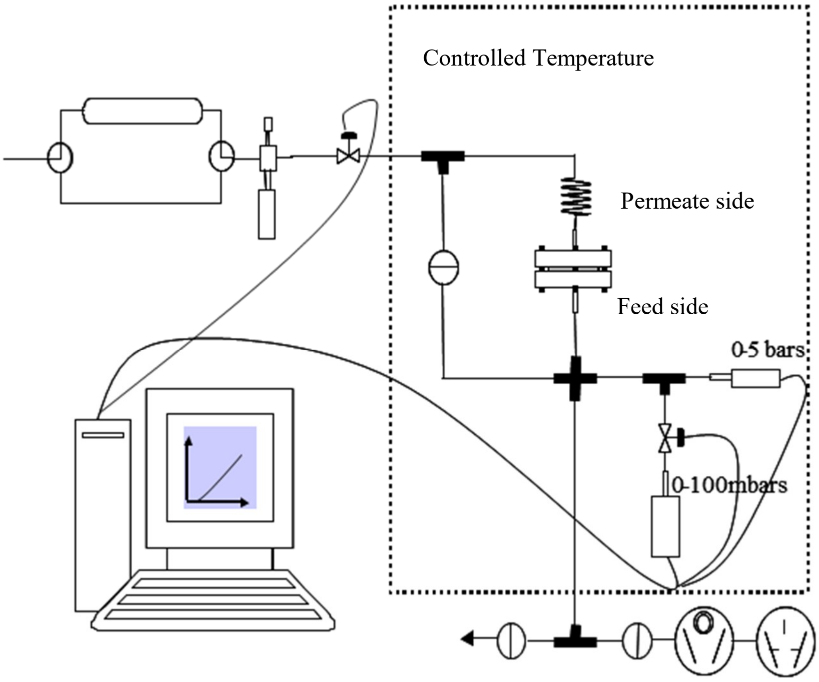

Figure S3. Schematic of the lab-scale experimental set-up used for single gas permeation measurements.
